# Advanced glycation end products as predictors of renal function in youth with type 1 diabetes

**DOI:** 10.1038/s41598-021-88786-4

**Published:** 2021-05-03

**Authors:** Josephine M. Forbes, Selena Le Bagge, Samuel Righi, Amelia K. Fotheringham, Linda A. Gallo, Domenica A. McCarthy, Sherman Leung, Tracey Baskerville, Janelle Nisbett, Adam Morton, Stephanie Teasdale, Neisha D’Silva, Helen Barrett, Timothy Jones, Jennifer Couper, Kim Donaghue, Nicole Isbel, David W. Johnson, Leigh Donnellan, Permal Deo, Lisa K. Akison, Karen M. Moritz, Trisha O’Moore-Sullivan

**Affiliations:** 1 Mater Research Institute, The University of Queensland, TRI, 37 Kent Street, Brisbane, QLD 4102 Australia; 2 School of Biomedical Science and Faculty of Medicine, The University of Queensland, St Lucia, QLD Australia; 3 Department of Medicine, University of Melbourne, Austin Health, Heidelberg, VIC Australia; 4 Mater Young Adults Health Centre, Mater Health Service, Brisbane, QLD Australia; 5 Telethon Kid’s Institute, Perth, WA Australia; 6 Robinson Research Institute, University of Adelaide, Adelaide, SA Australia; 7 Children’s Hospital at Westmead, Sydney, NSW Australia; 8 The Metro South and Ipswich Nephrology and Transplant Service (MINTS), Brisbane, QLD Australia; 9 Health and Biomedical Innovation, UniSA Clinical and Health Sciences, University of South Australia, Adelaide, SA Australia; 10 Child Health Research Centre, The University of Queensland, South Brisbane, QLD Australia

**Keywords:** Type 1 diabetes, Diabetes complications

## Abstract

To examine if skin autofluorescence (sAF) differed in early adulthood between individuals with type 1 diabetes and age-matched controls and to ascertain if sAF aligned with risk for kidney disease. Young adults with type 1 diabetes (*N* = 100; 20.0 ± 2.8 years; M:F 54:46; FBG-11.6 ± 4.9 mmol/mol; diabetes duration 10.7 ± 5.2 years; BMI 24.5(5.3) kg/m^2^) and healthy controls (*N* = 299; 20.3 ± 1.8 years; M:F-83:116; FBG 5.2 ± 0.8 mmol/L; BMI 22.5(3.3) kg/m^2^) were recruited. Skin autofluorescence (sAF) and circulating AGEs were measured. In a subset of both groups, kidney function was estimated by GFR_CKD-EPI CysC_ and uACR, and DKD risk defined by uACR tertiles. Youth with type 1 diabetes had higher sAF and BMI, and were taller than controls. For sAF, 13.6% of variance was explained by diabetes duration, height and BMI (*P*_model_ = 1.5 × 10^–12^). In the sub-set examining kidney function, eGFR and sAF were higher in type 1 diabetes versus controls. eGFR and sAF predicted 24.5% of variance in DKD risk (*P*_model_ = 2.2 × 10^–9^), which increased with diabetes duration (51%; *P*_model_ < 2.2 × 10^–16^) and random blood glucose concentrations (56%; *P*_model_ < 2.2 × 10^–16^). HbA_1C_ and circulating fructosamine albumin were higher in individuals with type 1 diabetes at high versus low DKD risk. eGFR was independently associated with DKD risk in all models. Higher eGFR and longer diabetes duration are associated with DKD risk in youth with type 1 diabetes. sAF, circulating AGEs, and urinary AGEs were not independent predictors of DKD risk. Changes in eGFR should be monitored early, in addition to uACR, for determining DKD risk in type 1 diabetes.

## Introduction

The presence of kidney disease (DKD) is the strongest predictor of mortality in individuals with diabetes^1^. In type 1 diabetes, it is increasingly appreciated that future risk for DKD and cardiovascular disease (CVD) may be evident as early as adolescence^2^, exacerbated by difficulties in maintaining adequate glycemic control at that time^3^. Risk for DKD is defined as an increase in urinary albumin excretion during puberty, preceding micro- and macroalbuminuria, as seen in adolescents with type 1 diabetes in the upper third of urinary albumin excretion^4^. However, best practice regimens for adults, targeting hypertension and dyslipidemia were ineffective at preventing microalbuminuria over a few years, in a Phase III clinical trial in adolescents with type 1 diabetes^4^. This suggests that other pathological factors may be at play during the early development of DKD.

Despite there being increasing scrutiny of childhood and adolescence, there is a paucity of data available examining risk for DKD in young adults with type 1 diabetes prior to the onset of chronic complications. These young people are often lost to follow up in the transition from pediatric to adult clinical care^5^, and commonly re-present later in adulthood with established diabetes complications, including DKD^6,7^. Understanding disease pathogenesis in young adults is also pertinent for type 2 diabetes given the growing number of children, adolescents and young adults diagnosed and their commonly accelerated progression to DKD in the context of adverse cardiovascular risk profiles^8^. Indeed, approximately 50% of individuals with type 1 diabetes develop DKD but this may be as high as 70%^9^.

The use of skin collagen associated advanced glycation end products (AGEs) to predict risk for diabetes complications including DKD comes from early studies by the Diabetes Control and Complications Trial/Epidemiology of Diabetes Interventions and Complications Trial (DCCT/EDIC) study group^10^. Here, greater accumulation of skin collagen AGEs, such as carboxymethyllysine (CML), predicted faster progression to DKD in young people with type 1 diabetes aged 20–30 years^10^. These quantitative studies of punch skin biopsies have evolved into non-invasive skin autofluorescence (sAF), a tool for assessing AGE-associated disease risk^11^. Previous cross-sectional^12–14^ and follow up studies^15^ in children, adolescents (< 18 years) and older adults (50–65 years) with diabetes have demonstrated that increases in sAF are an independent predictor of micro and macrovascular disease. AGE burden in diabetes can also be assessed using circulating or urinary AGE concentrations. Studies in adolescents and young people have mostly shown increases in circulating^16,17^ or urinary AGE concentrations^18^ with diabetes, but no independent association with diabetes complications^19,20^.

Hence, the first objective of this study was to examine if skin autofluorescence (sAF) differed in early adulthood (15–25 years of age) between individuals with type 1 diabetes and age-matched controls. The second objective was to ascertain if sAF aligned with risk for kidney disease, defined by the presence of type 1 diabetes and urinary albumin excretion tertile.

## Methods

### Study design and participants

The full cohort with diagnosed type 1 diabetes of greater than two years duration consisted of 100 adolescents/young adults (aged 15–25 years). Participants in this cohort did not have previously diagnosed diabetes complications, including kidney disease, neuropathy, retinopathy, and cardiovascular disease. Participants were recruited during their routine visit to the Transitional Care Clinic in Diabetes at the Mater Young Adult Health Centre. All participants that consented were included in the study. Exclusion criteria were (1) uncontrolled diabetes (defined as HbA_1C_ > 9.5%) or two episodes of ketoacidosis in the preceding 12 months, (2) history of severe family hypercholesterolemia, (3) previous myocardial infarction, stroke or pre-existing kidney disease, (4) pregnancy, (5) any existing medication other than insulin, (6) autoimmune diseases including uncontrolled coeliac disease, Addison’s disease, any congenital condition resulting in insulin dependent diabetes, and/or (7) diagnosed eating disorders.

The full control cohort (*N* = 299) was recruited from an undergraduate biomedical science course at the University of Queensland in 2018 and 2019. All students who completed the practical unit and who consented were included. The absence of diabetes was confirmed by a 2 h glucose measurement during a 75 g OGTT. OGTT was performed following standard procedure, with a 75 g/300 mL glucose load followed by blood glucose measurements at 30, 60, and 120 min after the glucose load^21^. Blood glucose was measured using a glucometer. A finger prick blood sample (~ 100 μl) was collected from a randomly selected group of control individuals (Renal Sub-set; *N* = 49) and was used for the eGFR measurement.

### Data and sample collection

Skin autofluorescence was assessed using an autofluorescence reader (mu AGE Reader; DiagnOptics, Groningen, the Netherlands) on a 4mm^2^ clean skin surface at the volar side of the forearm using a single device at both sites^11^. Four University students in the control group who had originally consented, were excluded due to documented use of tanning products or sunscreen which was not cleanly removed by ethanol wipes. Urinary AGEs were measured using LC–MS/MS as previously described^22^.

Data obtained from individuals at recruitment included chronological age, sex, height, weight and BMI. Height (cm) was measured using a mounted measuring stick and weight (kg) using standard scales. Body mass index (BMI) was calculated as weight (kg) divided by height squared (m^2^). For individuals with type 1 diabetes, age at diagnosis, diabetes duration, mean systolic blood pressure and a morning non-fasted and second void urine sample were also obtained.

### Biochemistry, renal function and risk for DKD

HbA_1C_ and random blood glucose were assessed using point of care devices. Quantikine ELISA kits were used for the measurement of serum cystatin C (R&D systems, Minneapolis, USA) according to the manufacturer’s instructions. Estimated GFR was calculated from serum cystatin C using the Chronic Kidney Disease Epidemiology Collaboration eGFR CKD-EPI-CysC equation, if serum cystatin C ≤ 0.8 mg/mL eGFR = 133 × min(S_cys_/0.8, 1)^−0.499^ × 0.996^Age^ × 0.932 [if female], and if serum cystatin C > 0.8 mg/mL eGFR = 133 × min(Scys /0.8, 1)^−1.328^ × 0.996^Age^ × 0.932 [if female]. In individuals with type 1 diabetes, the second morning urine void was collected for uACR and measured by the Mater Pathology routine laboratory. In addition to this uACR, we utilised the uACR measurements from two previous clinic visits to calculate mean urinary ACR values and define tertiles of ACR for each of the renal study participants based on previous studies^2,23^. Individual risk for DKD was allocated as: 0 = no diabetes (Control, 49 subjects), 1 = diabetes + lowest uACR tertile (Low Risk uACR ≤ 0.66 mg/mmol; 27 subjects), 2 = diabetes + middle uACR tertile (Medium Risk uACR = 0.67–1.16 mg/mmol; 29 subjects) or 3 = diabetes + upper uACR tertile (High Risk uACR ≥ 1.17 mg/mmol; 33 subjects). Albuminuria was defined according to international guidelines as uACR > 2.5 mg/mmol in males and > 3.5 mg/mmol in females.

### Statistical analyses

Data were expressed as mean ± SD or median (interquartile range) unless otherwise stated. Normality testing (Shapiro–Wilk) was performed on all data. Parametric data were analysed by one-way ANOVA with Tukey’s post hoc for multiple comparisons. Non-parametric data were analysed by Kruskal–Wallis and Dunn’s post hoc. Univariate modelling with Holm’s correction was used to determine interdependence of variables in the two cohorts. General linear models were used to examine the associations between sAF and were sequentially adjusted for covariates identified by univariate analyses in the full sAF cohort. Risk for DKD in the renal sub-study was also studied using general linear modelling.

### Ethics approval statement

This research protocol was approved by the Human Research Ethics committees of Mater Misericordiae Limited (Approval: HREC_15_MHS_35T1D) and the University of Queensland, Brisbane, Australia (Approval: 2016-02-066-PRE-3; UQ 2015-000-958). All investigation was conducted according to the principles of the Declaration of Helsinki.

### Patient consent statement

Written informed consent was obtained from all participants and if under 18 years of age, from their legal guardian in addition to the participant assent, prior to inclusion in the study.

## Results

**Figure 1:**
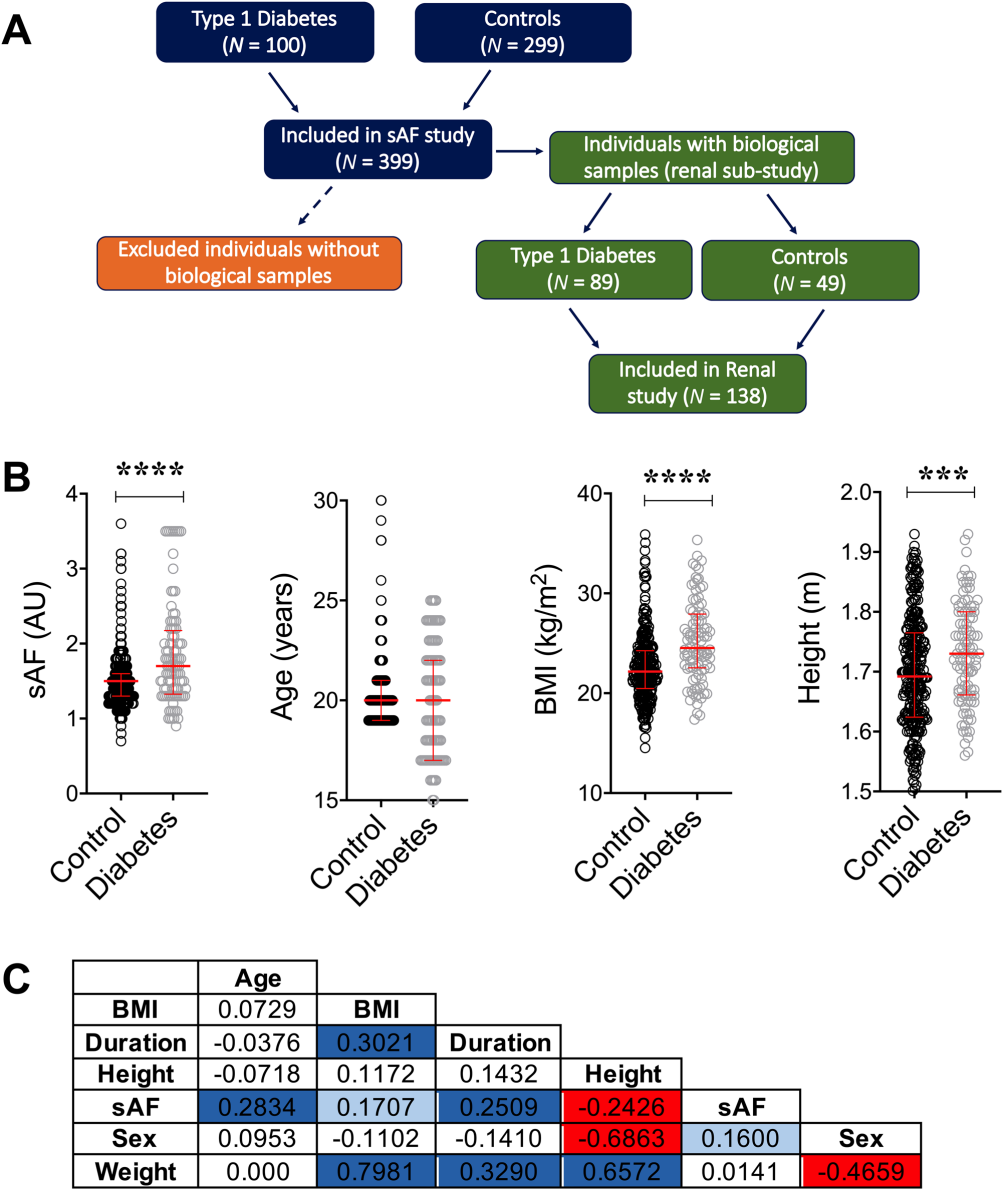
Overview of study design and baseline summary of the full cohort. (**A**) Flow chart of participant numbers. (**B**) Baseline characteristics of participants in the Full cohort for control individuals without diabetes (*N* = 299) and for individuals with type 1 diabetes (*N* = 100; Median IQR). ***P ≤ 0.001, ****P ≤ 0.0001 versus Control (No diabetes). (**C**) Spearman’s univariate correlation matrix corrected by Holm’s method. Significant *r* coefficients for positive associations are shown in dark blue—*P* ≤ 0.001 and light blue *P* ≤ 0.01 and significant negative relationships in red—*P* ≤ 0.001. *BMI* body mass index, *sAF* skin autofluorescence, Duration, diabetes duration.

Recruited individuals (Fig. 1A) were, on average, 20 years of age (Table 1). Baseline characteristics did not differ between the full sAF cohort and the Renal sub-set (Table 1). Those with diabetes were taller, with greater BMI, random blood glucose concentrations and sAF and with median diabetes duration of 10 years (Table 1, Fig. 1B). Holm’s corrected Spearman’s correlations in the full sAF and renal sub-set showed that sAF was positively associated with age, BMI and diabetes duration and negatively correlated with height (Fig. 1C). The duration of diabetes was also related to weight and consequently BMI (Fig. 1C).

**Table 1:** Baseline clinical and anthropometric characteristics.

Parameters	Full cohort (sAF) Control (*N* = 299)	Full cohort (sAF) Type 1 diabetes (*N* = 100)	Renal sub-set Control (*N* = 49)	Renal sub-set Type 1 diabetes (*N* = 89)
Age (years)	20 (3)	20 (5)	20 (2)	20 (4.5)
Sex (*N*, [% female])	299 (58)	100 (46)	49 (57)	89 (47)
Height (m)	1.69 (0.14)	1.73 (0.14)***	1.70 (0.15)	1.73 (0.14)**
Weight (kg)	64.3 (15.6)	75.0 (17.9)****	62.8 (15.5)	75.0 (18.2)****
BMI (kg/m^2^)	22.2 (3.8)	25.5 (5.4)****	21.9 (2.9)	24.3 (5.4)****
Random BG (mmol/L)	5.60 (1.25)^§^	12.15 (7.03)****	5.55 (0.93)	12.20 (6.30)****
Diabetes Duration (years)	0	10.0 (7.5)	0	10.0 (8.0)
HbA_1C_ %; [mmol/mol]	nd	8.2 (1.3);[66.1(12.8)]	nd	8.2 (1.2);[66.1(12.6)]

Data are median (IQR) or *N* (%). Participants included in the renal sub-set groups were from the full cohort who had biological samples taken (as per Fig. 1A). nd—not determined. Comparisons within full cohorts or sub-sets were by two-tailed Mann–Whitney Testing. Proportions were analysed by Fisher’s Exact test. **P < 0.01 versus control counterpart; ***P < 0.001 versus control counterpart; ****P < 0.0001 versus control counterpart; §*N* = 74 for random BG. BG—blood glucose.

General linear modelling for sAF with sequential addition of covariates, is shown in Table 2 for the full cohort. In Model 1 (Full cohort; *N* = 399 participants), diabetes duration and BMI were significant independent positive predictors, whilst height was a negative independent predictor of sAF (Adjusted *r*^*2*^ = 0.14, *P* = 1.47 × 10^–12^). This model was not appreciably improved by the addition of age and sex (Model 2; Adjusted *r*^*2*^ = 0.14, *P* = 8.77 × 10^–12^).

**Table 2:** General linear modelling for sAF predictors in the full cohort and DKD risk in the renal sub-study.

	β	SE	*P*_variable_	*P*_*m*odel_ *r*^*2*^_*adj*_
**Dependent: sAF**				
Model 1				
Diabetes duration	0.0250	0.0049	5.08 × 10^–7^***	1.47 × 10^–12^
Height	-1.2972	0.2682	1.93 × 10^–6^***	
BMI	0.0200	0.0066	0.0025**	0.1356
Model 2				
Diabetes duration	0.0248	0.0049	6.27 × 10^–7^***	8.77 × 10^–12^
Height	-1.2367	0.3613	6.88 × 10^–4^***	0.1372
BMI	0.0185	0.0066	0.0053**	
Age	0.0203	0.0124	0.1022	
Sex	0.0078	0.0695	0.9099	
**Dependent: DKD Risk**				
Model 1				
eGFR	0.0235	0.0038	5.53 × 10^–9^ ***	2.2 × 10^–9^
sAF	0.3259	0.1573	0.0402*	0.2446
Model 2				
Diabetes duration	0.1049	0.0122	2.42 × 10^–14^***	< 2.2 × 10^–16^
eGFR	0.0130	0.0033	1.2 × 10^–4^***	0.5077
sAF	0.0303	0.1310	0.8928	
Model 3				
Diabetes duration	0.0906	0.0124	2.42 × 10^–11^***	< 2.2 × 10^–16^
Random BG	0.0705	0.0173	7.78 × 10^–5^***	0.5594
eGFR	0.0097	0.0032	3.13 × 10^–3^**	
sAF	0.0161	0.1312	0.9023	
Model 4				
Diabetes duration	0.0872	0.0128	3.32 × 10^–10^***	< 2.2 × 10^–16^
Random BG	0.0699	0.0184	2.19 × 10^–4^***	0.5549
eGFR	0.0099	0.0033	3.12 × 10^–3^***	
Height	0.7168	1.0625	0.5011	
sAF	0.0088	0.1392	0.9499	
Sex (M)	-0.1667	0.1857	0.3711	

For full cohort—Control, *N* = 299; Diabetes, *N* = 100 individuals. For renal sub-study—Control, *N* = 49; Diabetes, *N* = 89 individuals. DKD risk is defined on a scale of 0–3, where 0 = No diabetes; 1 = type 1 diabetes and lowest uACR tertile; 2 = type 1 diabetes and middle uACR tertile and 3 = type 1 diabetes and highest uACR tertile. *SE* standard error, *sAF* skin autofluorescence, *BG* blood glucose, *eGFR* estimated glomerular filtration rate. *P < 0.05; **P < 0.01; ***P ≤ 0.001.

**Figure 2:**
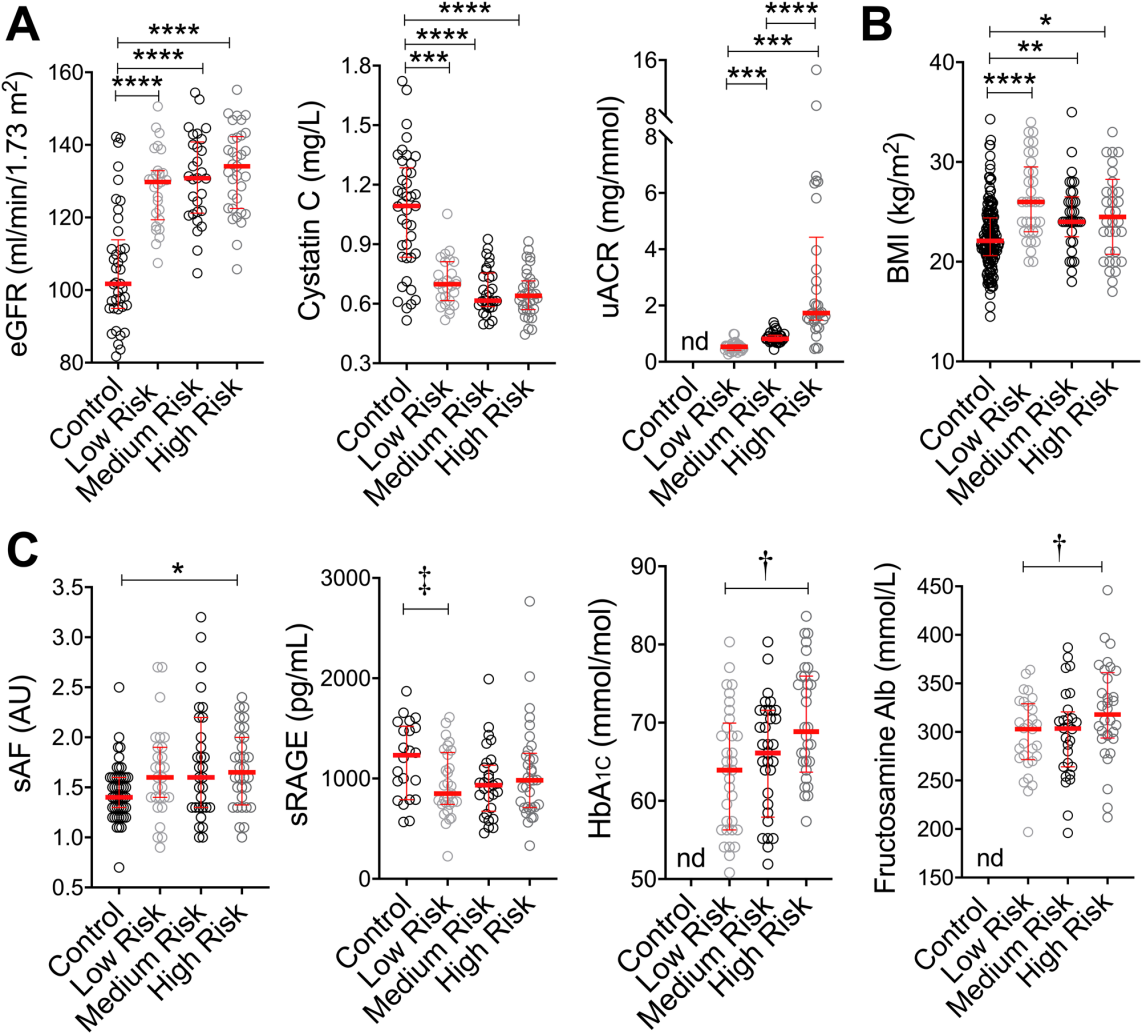
Advanced Glycation end products (AGE) and risk for diabetic kidney disease (DKD) risk in the Renal Sub-set. Characteristics of control individuals without diabetes (*N* = 49) and individuals with type 1 diabetes stratified by risk for DKD using tertiles of urinary albumin:creatinine ratio (uACR; Low Risk , *N* = 27; Medium Risk, *N* = 29; High Risk, *N* = 33. (**A**) Measurement of renal function using eGFR was calculated by the CKD-EPI_CYS_ formula, serum cystatin C (mg/L) for all participants and mean uACR in those with diabetes. (**B**) BMI (**C**) Assessment of AGE burden using sAF (skin autofluorescence), plasma soluble RAGE concentrations and the circulating glycated proteins fructosamine albumin and HbA_1C_. Data are median (IQR). *P < 0.05, **P ≤ 0.01, ***P ≤ 0.001, ****P ≤ 0.0001 versus No diabetes (Control); ^†^*P* < 0.05 versus Low Risk. ^‡^*P* < 0.05 versus Control. nd – not determined.

In those who had biological samples taken (Renal sub-set; Suppl. Table 1; Fig. 1; *N* = 148), individuals with type 1 diabetes had significantly higher eGFR and lower serum cystatin C and progressive increases in uACR, as DKD risk increased (Fig. 2A), as predicted. In this Renal sub-set, 12.8% of males and 10.3% of females with type 1 diabetes had microalbuminuria. BMI was greater in young individuals with type 1 diabetes (Fig. 2B).

When examining AGE burden in the renal sub-set, sAF remained greater, as per the full cohort, in individuals with type 1 diabetes versus control (Fig. 2C, P = 0.0004) and was modestly higher in subjects at greatest risk for DKD. Using general linear modelling with sequential addition of covariates, Model 1 explained 17.3% of variance in sAF (Adjusted *r*^*2*^ = 0.17, *P* = 1.0 × 10^–6^), where BMI, diabetes duration and height were independent variables (Suppl. Table 2). This was strengthened by the addition of random BG in Model 2 (Adjusted *r*^*2*^ = 0.20, *P* = 3.6 × 10^–7^) but not further improved by the addition of age and sex in Model 3 (Suppl. Table 2; Adjusted *r*^*2*^ = 0.20, *P* = 1.0 × 10^–6^).

Serum soluble RAGE (sRAGE) concentrations differed between control and low (*P* < 0.05), but not medium or high risk individuals (Fig. 2C; *P* = 0.069). Overall, circulating sRAGE was lower in those youth with type 1 diabetes (973 ± 349 pg/ml) versus controls (1184 ± 379 pg/ml, *P* = 0.0245). The glycated proteins HbA_1C_ and fructosamine albumin, each indicative of longer-term glycaemic control, were greatest in the high versus low risk tertile for DKD (Fig. 2C). Urinary excretion of both the protein bound and free AGEs, MG-H1 and CML, did not differ between risk groups (Suppl. Fig. 1). A Holm’s corrected Spearman’s correlation matrix showed that sAF was positively related to age, BMI and risk for DKD in the renal sub-set. Risk for DKD was positively related to BMI, diabetes duration, eGFR and random BG (Suppl. Fig. 1A). Soluble RAGE concentrations were negatively associated with BMI and diabetes duration (Suppl. Fig. 1A).

Using univariate analysis in just those individuals with type 1 diabetes, sAF was positively associated with age (*r* = 0.37, *P* = 0.0008), diabetes duration (*r* = 0.22, *P* = 0.047 and BMI (*r* = 0.27, *P* = 0.017) and height (*r* = 0.27, *P* = 0.017), but not with any indices of glycaemic control, including HbA_1C_ or other AGE measurements. uACR was most strongly related to plasma sRAGE (*r* = 0.30, *P* = 0.0068) and urinary MG-H1 concentrations (*r* = 0.33, *P* = 0.0025) in individuals with type 1 diabetes. These associations did not persist following Holm’s adjustment. eGFR remained independent to all patient variables collected in this sub-analysis of individuals with type 1 diabetes. Indeed, even a model containing uACR, diabetes duration, age, sex, BMI, HbA_1C_, SBP, random BG and total cholesterol explained only ~ 4% of variance in eGFR in youth with type 1 diabetes and was not significant (Adjusted *r* = -0.0375, *P*_*model*_ = 0.7293). Addition of urinary CML, MG-H1, sAF and circulating sRAGE did not appreciably improve the model.

General linear modelling for DKD risk with sequential addition of covariates in the renal sub-study, is shown in Table 2. Firstly, eGFR predicted 23% of the variance in DKD risk (Adjusted *r* = 0.25, *P* = 2.3 × 10^–9^). In Model 1 (Adjusted *r* = 0.25, *P* = 2.2 × 10^–9^), eGFR (*P* = 5.5 × 10^–9^) and sAF (*P* = 0.04) were significant independent predictors of DKD risk. With additional adjustment for diabetes duration (Table 2; Model 2, Adjusted *r* = 0.51, *P* < 2.2 × 10^–16^), the ability of the model to predict DKD risk variance doubled to greater than 50%. eGFR remained an independent predictor of DKD risk in this model (*P* = 1.2 × 10^–4^ ). With the addition of random blood glucose in Model 3, diabetes duration and eGFR remained independent predictors of DKD risk but the prediction of DKD risk by this model only modestly increased (Table 2, Model 3; Adjusted *r* = 0.56, *P* < 2.2 × 10^–16^). With the addition of height and sex in Model 4, diabetes duration, FBG and eGFR remained independent predictors of DKD risk, but overall the prediction of DKD risk did not appreciably increase (Table 2, Model 4; Adjusted *r* = 0.56, *P* < 2.2 × 10^–16^). The addition of circulating sRAGE and urinary AGEs did not alter the explained variance in DKD risk in any of these models.

## Discussion

In the present study, biomarkers of DKD related to advanced glycation were investigated in youth. Greater eGFR, random blood glucose concentrations and diabetes duration were independent markers for DKD, accounting for > 55% of variation in this young renal cohort. Risk prediction models were not further improved by the addition of skin autofluorescence (sAF), nor other AGE or sRAGE measurements. In our larger population where biological samples were not available, diabetes duration, BMI and height were significant independent predictors of sAF. Height may have been influenced by a modestly greater proportion of males and was not related to sex in univariate analysis. However, the inverse relationship between sAF and height may also be explained by previous studies suggesting that after diabetes diagnosis, those adolescents with the poorest glycaemic control, end up with the greatest deficit in final height^24,25^. Certainly those with poorer glycaemic control would not only be more likely to have higher sAF but also greater risk for chronic complications^25^. However, together these covariates explained only ~ 13% of sAF variance in our entire population and ~ 20% in the renal sub-study with the addition of random blood glucose concentrations. This implies that other major factors are contributing to sAF in young adults at the age groups we are examining that were not identified in this study. Indeed, while sAF is known to increase in parallel with age and diabetes duration in young people^26^, height has not been previously identified as a predictor of sAF in this group.

sAF was significantly greater in young people with type 1 diabetes compared to controls, which is consistent with previous work in adults^27–29^ and in children and adolescents^26^. However, when considering the renal sub-study who had biological samples taken, sAF was an independent predictor of DKD risk in a model that included eGFR. Certainly in a previous study, sAF did increase according to chronic kidney disease stage^30^ and within the DCCT/EDIC study, a slightly older cohort than ours, predicted the development of both micro- and macrovascular disease, including kidney disease. With addition of diabetes duration to our models, where sAF has shown dependency in previous studies^12^, the variability in DKD risk significantly improved to > 50%. Indeed, diabetes duration has been consistently shown as an important determinant of complication risk in diabetes as shown in the present study.

Glycaemic control is the most commonly targeted risk factor for DKD, but target control is notoriously difficult to achieve in youth^31^. Certainly, addition of random blood glucose concentrations to diabetes duration, eGFR and sAF explained more of the variance in DKD risk in the renal cohort (up to 56%). Other measures of glycaemic control, namely fructosamine albumin, which are both glycated proteins but early advanced glycation adducts, were elevated with diabetes to a greater degree in high risk individuals. Unfortunately, these measurements were not available in our control subjects. However, adding these variables did not appreciably improve DKD risk prediction in individuals with type 1 diabetes. It is possible that youth in the present study were assessed too early in the course of disease for measures of advanced glycation, such as sAF, to differentiate those at higher risk for DKD. Indeed, we had specifically excluded youth with previously diagnosed kidney disease. Further, most previous studies demonstrating that sRAGE^32^ and AGEs^33^ as independent predictors of DKD or macrovascular disease in diabetes, were performed in older people. Interestingly, in the Adolescent Type 1 Diabetes Cardio-Renal Intervention Trial (AdDIT), HbA_1c_ measurements were remarkably similar among groups and did not align with greater DKD risk^34^. However, in recent follow up of these adolescents from AdDIT at a similar age to our cohort, HbA_1c_ concentrations were significantly higher in those individuals previously allocated to the upper tertile of uACR, and predicted to be at greatest risk for DKD^23^. This agrees with HbA_1C_ concentrations in our youth with type 1 diabetes, which were higher in those at greatest risk for DKD. Additionally, in young people with type 1 diabetes, complications including DKD, often initially progress without changes in conventional risk factors, such as HbA_1C_^35^. This suggests that the development of DKD in the early stages may not be as reliant on poor glycaemic control as previously thought and abnormalities in uACR may, in fact precede worsening glycaemic control.

Surprisingly, a model which explained eGFR variance (to greater than 5%) in individuals with type 1 diabetes using sequential addition of all of the covariates collected, could not be found in this cohort of 15–25 year olds. This suggests that other factors may be driving eGFR changes at this early time point in the development of diabetic kidney disease. Furthermore, youth with type 1 diabetes had significantly higher eGFR values compared to controls, indicating hyperfiltration. This outcome is consistent with the recent follow up of the AdDIT cohort, in which eGFR was also greater in those young people in the highest tertile of uACR^23^. Hyperfiltration is present in the majority of young people with type 1 diabetes including in the present study, and likely precedes progressive decline in eGFR^36^. Indeed, a meta-analysis of 12 studies, which included youth and adults, found that people with the most significant hyperfiltration were more likely to progress to DKD^37^. In young people and adults with hyperfiltration, a greater increase in uACR was also present over the 8 year follow up period, compared to those with normal glomerular filtration^38^. However, other studies in young people have not identified a link between hyperfiltration and microalbuminuria^39^. In young people in the Oxford Regional Prospective Study, the cumulative prevalence of microalbuminuria was 50.7% after 19 years of diabetes^2^. This is approximately 10 years later than the cohorts examined here, where ~ 12% of individuals with diabetes had microalbuminuria despite the present study excluding individuals with pre-existing kidney disease requiring medication. It is important to note however that rates of microalbuminuria do vary in adolescents in the general population, where rates of between 3.3 and 14% have been reported^40^. DKD is known to develop progressively with some subjects frequently reverting to normoalbuminuria, suggesting that subtle changes in eGFR are important to monitor alongside uACR in youth with type 1 diabetes.

In addition to eGFR, BMI was also significantly higher in youth with type 1 diabetes compared to controls. However risk did not vary among DKD risk tertiles, which is in agreement with the recent AdDIT follow up study, where BMI also did not significantly differ among uACR tertiles^23^. In the present study, BMI was positively associated with sAF and was an independent predictor of sAF in a model that included diabetes duration and height. This positive association has been previously described in adults with^11,41^ and without diabetes^42^. The increased AGE accumulation in people with higher BMI could be a result of increased dietary intake of AGEs or oxidative stress^43^, but some factors known to affect sAF were not assessed in the present study. Indeed, lower sRAGE concentrations in our youth with type 1 diabetes, could adversely impact AGE clearance. Lower sRAGE also consistently associates with greater BMI and a causal link between obesity and DKD in adults with type 1 diabetes has been demonstrated^44^.

### Limitations

Limitations of the present study include the absence of control data for eGFR, HbA_1C_, fructosamine, uACR, and urinary AGEs. Furthermore, numerous factors are known to influence sAF levels, which were not controlled in this study, including HbA_1C_ (for the control group), alcohol and coffee consumption and smoking^42^. Importantly, ethnicity was not recorded, which is known to influence sAF. The timing and AGE content of a participant’s previous meal could also have conceivably increased sAF post-prandially^45^. However, the influence of these factors on sAF in this cohort is likely to be relatively small when compared with the presence of diabetes per se. Additionally, the limitations of the AGE reader used to measure sAF have been previously described, but it has been validated as a reliable surrogate marker of AGE burden in an adolescent population^46^. Finally, given that this was a cross-sectional observational study, we did not have follow up data for these individuals to determine if/when they developed diabetic kidney disease.

## Conclusion

Taken together, these studies suggest that greater eGFR and diabetes duration in youth with type 1 diabetes without previously diagnosed complications are markers of DKD risk, which are not improved by measurement of sAF nor other markers of AGE burden. However, there may be some utility for the routine measurement of early glycation adducts such as fructosamine albumin, in addition to more routine HbA_1C_. Further, early changes in eGFR during diabetes should be monitored alongside uACR to better stratify those young people with type 1 diabetes at greatest risk for DKD and CVD. This is of significant interest, as current methods for prediction of early DKD are poor, which often delays appropriate clinical management until more advanced complications develop^47^. Additional longitudinal studies are required to better establish risk factors for GFR decline, since this could not be ascertained in this study and appeared independent of uACR.

## Supplementary information

Supplementary Information.
