# Supplementary material for: Advanced glycation end products as predictors of renal function in youth with type 1 diabetes
Source: Sci Rep. 2021 May 3;11:9422. doi: 10.1038/s41598-021-88786-4 (PMC8093271; doi:10.1038/s41598-021-88786-4)
Supplement: Supplementary file 1 — Supplementary Information. [file 41598_2021_88786_MOESM1_ESM.docx]

**Supplementary Material for: “Advanced glycation end products as predictors of renal function in youth with type 1 diabetes”**

**Short running title:** AGEs and renal function in youth with T1D

Josephine M. Forbes PhD^1,2,3^*, Selena Le Bagge BAdvSc (Hons)^1,2^*, Samuel Righi MD^1^, Amelia K. Fotheringham PhD^1,2^, Linda A. Gallo PhD^1,2^, Domenica A. McCarthy BSc^1^, Sherman Leung PhD^1,2^, Tracey Baskerville BNurs^1,4^, Janelle Nisbett MD^4^, Adam Morton MD^4^, Stephanie Teasdale MD^4^, Neisha D’Silva MD^4^, Helen Barrett MD^1,4^, Timothy Jones MD^5^, Jennifer Couper MD^6^, Kim Donaghue MD^7^, Nicole Isbel MD^2,8^, David W. Johnson MD ^2,8^, Leigh Donnellan BSc (Hons)^9^, Permal Deo PhD^9^, Lisa K. Akison PhD^2,10^, Karen M. Moritz PhD^2,10^, Trisha O’Moore-Sullivan MD^1,4^

*Equal contribution.

**Affiliations:**

^1^Mater Research Institute - The University of Queensland, TRI, Brisbane, Queensland, Australia

^2^School of Biomedical Science and Faculty of Medicine, The University of Queensland, St Lucia, Queensland, Australia

^3^Department of Medicine, University of Melbourne, Austin Health, Heidelberg, Victoria, Australia

^4^Mater Young Adults Health Centre, Mater Health Service, Brisbane, Queensland, Australia

^5^Telethon Kid’s Institute, Perth, Western Australia, Australia

^6^Robinson Research Institute, University of Adelaide, Adelaide, South Australia, Australia.

^7^Children’s Hospital at Westmead, Sydney, New South Wales, Australia

^8^The Metro South and Ipswich Nephrology and Transplant *Service* (MINTS), Brisbane, Queensland, Australia

^9^Health and Biomedical Innovation, UniSA Clinical and Health Sciences, University of South Australia, South Australia, Australia

^10^Child Health Research Centre, The University of Queensland, South Brisbane, Queensland, Australia

| **Parameters** | **No diabetes**  (*N* = 49) | **Type 1 diabetes**  **Low Risk**  (*N* = 27) | **Type 1 diabetes**  **Medium Risk**  (*N* = 29) | **Type 1 diabetes**  **High Risk**  (*N* = 33) |
| --- | --- | --- | --- | --- |
| Age (years) | 20.33±1.82 | 20.44±2.65 | 19.97±2.65 | 20.09±3.05 |
| Sex (*N*, % female) | 49 (57) | 27 (37) | 29 (50) | 33 (49) |
| Height (m) | 1.69±0.10 | 1.74±0.08 | 1.73±0.09 | 1.73±0.09 |
| Weight (kg) | 64.1±11.4 | 80.2±14.5^****^ | 74.1±11.5^**^ | 77.2±21.2^**^ |
| BMI (kg/m^2^) | 22.5±3.3 | 26.4±4.3^****^ | 24.9±3.6^**^ | 25.0±5.4^*^ |
| Random BG (mmol/L) | 5.21±0.80 | 10.89±4.33^****^ | 11.49±3.90^****^ | 12.02±4.97^****^ |
| Diabetes Duration (years) | 0 | 9.74±5.08^****^ | 11.17±4.62^****^ | 10.91±5.64^****^ |
| HbA_1C_ %; (mmol/mol) | n.d. | 8.0±0.7; 63.4±7.9 | 8.2±0.8; 64.9±8.2 | 8.5±1.2; 68.9±10.4^†^ |

**Supplementary Table 1** – Baseline characteristics in the renal sub-set for control individuals without diabetes and for individuals with type 1 diabetes stratified for risk for DKD by tertiles of urinary albumin to creatinine ratio (uACR). n.d- not determined. **P* < 0.05; ***P* ≤ 0.01; *****P* ≤ 0.0001 vs no diabetes; ^†^ *P* < 0.05 vs low risk tertile. BG – blood glucose.

| **Dependent: sAF** | **β** | **SE** | ***P*_variable_** | ***P_m_*_odel_**  ***r^2^_adj_*** |
| --- | --- | --- | --- | --- |
| **Model 1**  BMI  Diabetes Duration  Height | 0.0336  0.0234  -1.5553 | 0.0103  0.0072  0.5095 | 1.36x10^-3^**  1.44x10^-3^**  2.70x10^-3^** | 1.02x10^-6^  0.1731 |
| **Model 2**  BMI  Height  Diabetes Duration  Random BG | 0.0369  -1.6025  0.0180  0.0203 | 0.0100  0.5077  0.0077  0.0112 | 3.22x10^-4^***  1.95x10^-3^**  0.0212*  0.0730 | 3.58x10^-7^  0.1982 |
| **Model 3**  BMI  Height  Random BG  Diabetes Duration  Age  Sex | 0.03586  -1.9937  0.0240  0.0160  0.0266  0.0996 | 0.0100  0.6743  0.0113  0.0077  0.0173  0.1187 | 4.89x10^-4^***  3.65x10^-3^**  0.0371*  0.0419*  0.1257  0.4026 | 9.96x10^-7^  0.2036 |

**Supplementary Table 2:** General Linear Modelling for sAF Predictors within the renal sub-set. (control, *N*=49; diabetes, *N*=89 individuals). SE - standard error; sAF - skin autofluorescence; BG - blood glucose. **P* < 0.05; ***P* ≤ 0.01; ****P* ≤ 0.001.


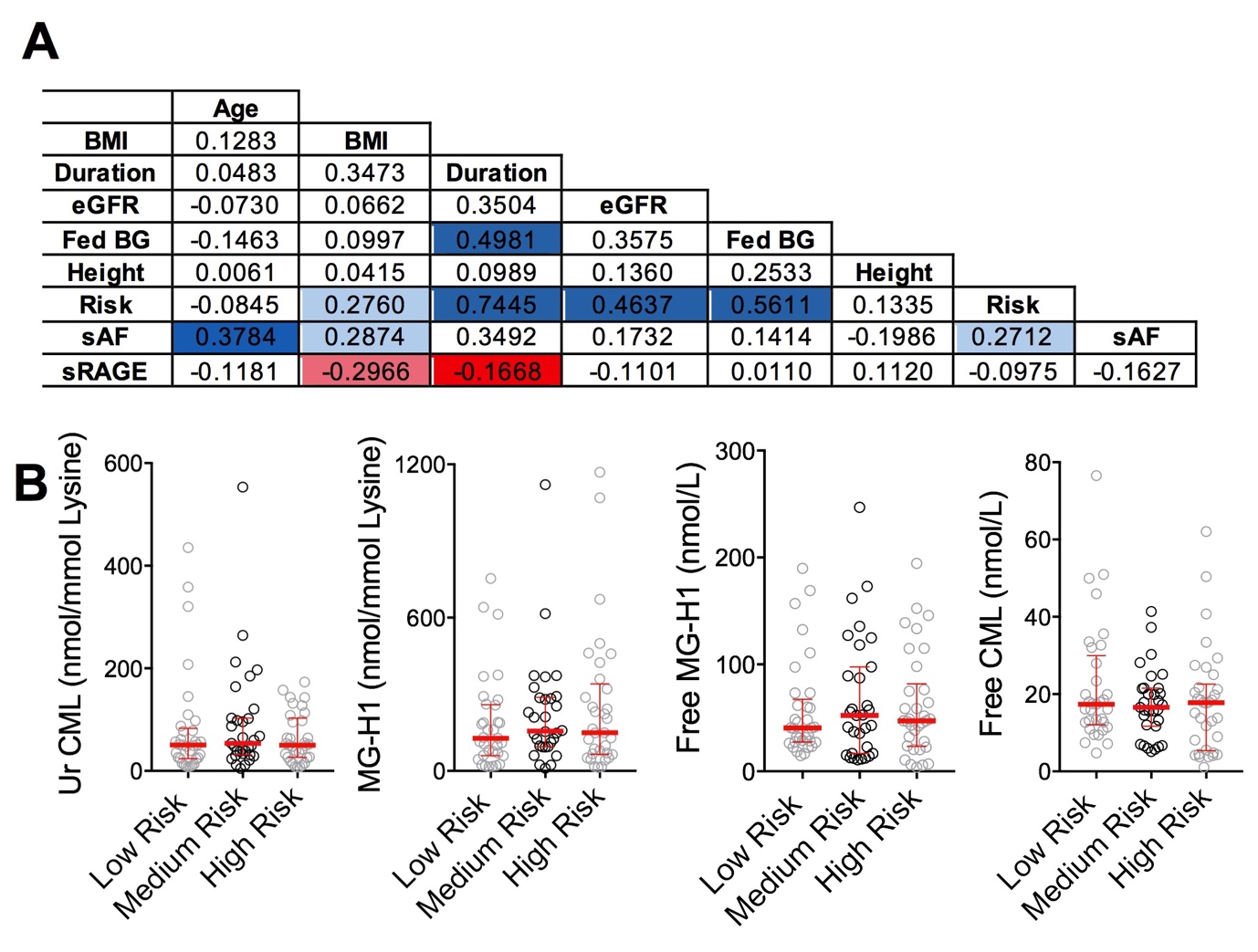


**Supplementary Figure 1: Spearman Correlation Matrix Assessment of the Renal sub-set**. Control individuals without diabetes (N=49) with Very Low Risk and in individuals with type 1 diabetes stratified for risk for diabetic kidney disease by tertiles of uACR (Low Risk, *N*=27; Medium Risk, *N*=29; High Risk, *N*=33. **A)** Spearman’s Univariate Correlation Matrix corrected by Holm’s Method. Significant positive associations are shown in dark blue - *P* ≤ 0.001 and light blue - *P* < 0.05 and significant negative relationships in red - *P* ≤ 0.001 and light Red - *P* < 0.05. **B)** Assessment of AGE burden using urinary CML (N^€^-carboxymethyllysine) and MG-H1 (methylglyoxal-derived hydroimidazolone) when protein bound (per mole of lysine) and free . eGFR – estimated glomerular filtration rate; BG – blood glucose; sAF – skin autofluorescence; sRAGE – soluble receptor for advanced glycation end products.
